# Survival Trends Associated With Histopathological Risk Factors in Oral Squamous Cell Carcinoma

**DOI:** 10.1155/ijod/8821799

**Published:** 2026-05-08

**Authors:** Shyam Sthanukrishnan, Smitha Sammith Shetty, Chetana Chandrashekar, Deepak Nayak, Raghu Radhakrishnan

**Affiliations:** ^1^ Department of Oral and Maxillofacial Pathology, Manipal College of Dental Sciences, Manipal Academy of Higher Education, Manipal, Karnataka, 576104, India, manipal.edu; ^2^ Department of Pathology, Kasturba Medical College, Manipal Academy of Higher Education, Manipal, Karnataka, 576104, India, manipal.edu; ^3^ Academic Unit of Oral Biology and Oral Pathology, Oman Dental College, P.O. Box 835, Mina Al Fahal, 116, Oman, odc.edu.om; ^4^ Academic Unit of Oral and Maxillofacial Medicine and Pathology, School of Clinical Dentistry, University of Sheffield, Sheffield, S10 2TA, UK, sheffield.ac.uk

**Keywords:** disease-free survival, histological risk, overall survival, risk stratification

## Abstract

**Background:**

Histopathological risk assessment models in oral squamous cell carcinoma (OSCC) have had varying degrees of success in stratifying patients, with models developed on retrospective cohorts being able to predict recurrence and metastasis. In spite of mounting evidence on their impact on survival outcomes, the incorporation of histopathological features, including perineural invasion (PNI) and the worst pattern of invasion, in staging and risk stratification is still lacking. Thus, our study aimed to assess and characterise histological prognostic indicators in OSCC, elucidate their significance in relation to patient prognosis, and develop a risk assessment model by correlating these indicators with survival parameters and risk stratification.

**Methods:**

A retrospective chart audit was performed, and histologic parameters were staged according to the American Joint Committee on Cancer’s 8th Edition Cancer Staging Manual and objectively scored based on the College of American Pathologists’ (CAP) Protocol 2018. Pearson’s chi‐square test and Kaplan–Meier survival analysis were carried out to identify the significant correlations between specific histopathological indicators and patient prognosis.

**Results:**

Higher grades of pattern of invasion were significantly associated with greater PNI and depth of invasion (*p* < 0.05). Depth of invasion was also significantly associated with extranodal extension (ENE) (*p* < 0.05). Survival analysis showed a clear trend with higher grades of the worst pattern of invasion and depth of invasion, as well as PNI, as possible independent predictors of poorer survival outcomes.

**Conclusion:**

The development of a risk stratification model based on these indicators provides a valuable tool for enhancing patient outcomes. Incorporation of detailed histopathological analysis into routine clinical decision‐making improves survival rates and treatment efficacy in OSCC patients.

## 1. Introduction

Head and neck squamous cell carcinoma (HNSCC) encompasses a group of malignancies that originate from squamous epithelial cells within the head and neck region [1]. HNSCC includes malignancies of the oral cavity lining, pharynx, larynx, paranasal sinuses and salivary glands [2]. According to GLOBOCAN 2020 statistics, HNSCC ranks as the seventh most common cancer worldwide, with an estimated 890,000 new cases annually, accounting for ~4.5% of all cancers globally [3].

Oral squamous cell carcinoma (OSCC) is a major subset of HNSCC, constituting up to 90% of all oral malignancies. OSCC accounts for approximately half of all HNSCC cases diagnosed annually [1]. While primary surgery with clear margins remains the mainstay of management, especially in early‐stage OSCC, 25%–45% of patients develop local recurrence [4]. The 5‐year survival rate drops from 45%–65% to 20% when there is regional lymph node metastasis, which is often observed in early‐stage OSCC with features such as poor differentiation or lymphatic invasion [5, 6].

Histopathological risk assessment models that are based on correlations between histopathological features and survival parameters have had varying degrees of success in stratifying patients based on risk models developed on retrospective cohorts that predict recurrence and regional metastasis [7]. A large cohort study like the one by Ebrahimi et al. [8] successfully proved the utility of incorporating depth of tumour invasion (DOI) in patient stratification, which was subsequently included as a staging criterion in the 8th Edition of the Staging Manual of the American Joint Committee on Cancer (AJCC 8), along with extranodal extension (ENE) [9, 10]. Although the College of American Pathologists (CAP) Protocol 2018 outlines reporting criteria and classification of histopathological features, including DOI and ENE, and in spite of mounting evidence on their impact on survival outcomes, their incorporation in staging and risk stratification is still lacking [11].

In our study, we sought to analyse the association between the various histopathological features, namely depth of invasion, ENE, perineural invasion (PNI) and worst pattern of invasion, first among themselves and subsequently with survival in terms of disease‐free and overall survival.

## 2. Materials and Methods

After obtaining the Institutional Ethical Committee approval, histopathologically confirmed cases of gingivobuccal OSCC classified according to the AJCC 8th edition criteria were included.

### 2.1. Study Population

The sample size of 85 was determined by assuming an exponential parametric survival distribution for gingivobuccal OSCCs, where events included either locoregional recurrence, distant metastasis or death.

Inclusion criteria:

• Histopathologically confirmed cases of OSCC.
• Cases treated with primary curative surgery with or without adjuvant therapy.
• Adequate follow‐up data of at least 1 year from the date of primary curative surgery.

Exclusion criteria:

• Tumours which are not OSCC.
• The OSCC of the sites of the oral cavity other than gingivobuccal mucosa.

### 2.2. Electronic Medical Records Data Extraction

After obtaining the requisite ethical clearance, cases meeting the inclusion and exclusion criteria were identified, and a retrospective chart audit of the clinicopathological and demographic details extracted from archival data and electronic medical records from 2012 to 2021 was performed.

### 2.3. Evaluation and Analysis of Histopathological Features

Four histopathological features, viz., depth of invasion, worst pattern of invasion, PNI and ENE, extracted from the medical records, were assessed based on the CAP 2018 protocol, and an objective scoring criteria was applied as shown in Table 1.

**Table 1:** Scoring criteria for histopathological parameters in oral squamous cell carcinoma.

Parameters	Diagnostic feature	Score
Anatomical location (AL)	Gingivobuccal complex	1
	Tongue	2
	Floor of the mouth	3
	Palate	4
Histological grade (HG)	Well‐differentiated	1
	Moderately differentiated	2
	Poorly differentiated	3
Nodal involvement (NI)	Present	1
	Absent	2
Margin status (MS)	Clear margins (>5 mm)	1
	Close margins (0–5 mm)	2
	Involved margins	3
Depth of invasion (DOI)	Not reported	0
	≤5 mm	1
	>5 mm	2
	>10 mm	3
Worst pattern of invasion (WPOI)	Not reported	0
	Type 1—pushing border	1
	Type 2—finger‐like border	2
	Type 3—large separate islands with more than 15 cells per island	3
	Type 4—small tumour islands with less than 15 cells per island	4
	Type 5—tumour satellites ≥1 mm from main tumour or closest tumour satellite	5
Perineural invasion (PNI)	Absent	1
	Present around small nerves	2
	Present around large nerves	3
	Not reported	0
Lymphovascular invasion (LVI)	Absent	1
	Present	2
	Not reported	0
Extranodal extension (ENE)	Absent	1
	Present	2
	Not reported	0
Lymphocyte host response (LHR)	Type I	1
	Type II	2
	Type III	3
	Not reported	0
Pathological T‐stage (pT)	T1	1
	T2	2
	T3	3
	T4	4
Pathological N‐stage (pN)	N0	0
	N1	1
	N2	2
	N3	3

### 2.4. Statistical Analysis

Pearson chi‐squared test of association was employed to analyse the association between the various histopathological features. Kaplan–Meier survival analysis was performed to evaluate the impact of histopathological features on survival in terms of overall and disease‐free survival. All statistical analyses were performed using R statistical software (Version 4.4.2).

## 3. Results

### 3.1. Study Group Selection

The retrospective chart audit showed 120 cases of OSCC that underwent primary curative surgery and had adequate follow‐up data. Out of these, 85 cases which had details of adverse histological features were subjected to association analyses.

### 3.2. Clinicopathological and Demographic Data

The patients’ ages ranged from 29 to 77 years (median = 53 years, interquartile range 45.5–60), and 97 (81%) were males and 23 (19%) were females. Histological grading of the tumours showed 87 cases (72.5%) of well‐differentiated SCC, 30 cases (25%) of moderately differentiated SCC and 3 cases (2.5%) of poorly differentiated SCC. Anatomically, 87 cases (72.5%) were from the gingivobuccal complex, 32 (26.67%) from the tongue and one from the hard and soft palate. Pathological staging based on 8th edition of the AJCC Cancer Staging Manual showed 43 cases presenting in stage T4, 17 cases in T3, 39 cases in T2 and 22 cases in T1 stage. Half of the cases presented at a late stage (T3/T4), stressing the importance of early detection (Table 2).

**Table 2:** Clinicopathological and demographic characteristics of the study population.

Clinicopathologic and demographic parameters		Summary statistics
Median age (in years)		53 (IQR = 45.5–60)
Gender		Males—97 (81%) Females—23 (19%)
Histological tumour grade	Well‐differentiated SCC	87 (72.5%)
	Moderately differentiated SCC	30 (25%)
	Poorly differentiated SCC	3 (2.5%)
Anatomical location	Gingivobuccal complex	87 (72.5%)
	Tongue	32 (26.67%)
	Hard and soft palate	1
pT stage	T1	21 (17.5%)
	T2	39 (32.5%)
	T3	17 (14.17%)
	T4	43 (35.83%)
pN stage	N0	60 (50%)
	N1	18 (15%)
	N2	19 (15.83%)
	N3	23 (19.17%)
Margin status	Clear margins (>5 mm)	95 (79.17%)
	Close margins (<5 mm)	11 (9.17%)
	Involved margins	14 (11.67%)

Abbreviations: IQR, interquartile range; SCC, squamous cell carcinoma.

### 3.3. Clinical Outcome

A total of 32 cases (26.7%) developed locoregional recurrence, 4 cases (3.3%) developed distant metastasis and 9 (7.5%) died during follow‐up owing to a tumour‐related issue. A total of 60 cases (50%) presented with Stage I or early‐stage disease with no nodal involvement (pT1N0/pT2N0). A total of 95 cases (79.2%) had clear surgical margins of more than 5 mm, 11 cases (9.2%) had close margins and 14 (11.7%) had involved margins which had either tumour deposits or dysplasia. Primary curative surgery was accompanied by supra‐omohyoid or extended supra‐omohyoid neck dissection in 100 patients (83%), modified radical neck dissection in 15 patients (12.5%) and no neck dissection in 5 patients (4%). Following surgery, patients were reviewed for the requirement of adjuvant therapy. A total of 60 (50%) patients were advised adjuvant radiotherapy alone, out of which 55 completed the treatment and 5 defaulted. A total of 33 patients (27.5%) were advised adjuvant chemoradiation, out of which 31 completed the treatment and 2 defaulted. The 3‐year locoregional control, distant control, disease‐specific survival and overall survival rates were 73.3%, 66.7%, 92.5% and 92.5%, respectively. The median follow‐up period was 606 days. Nodal involvement, margin status, histological tumour grade and pathological T and N stages did not significantly influence the probability of the development of locoregional recurrence or distant metastasis (Fisher’s exact test, *p* > 0.05).

### 3.4. Association Between Histological Features and Clinicopathological Data

The 85 cases studied for association among the histopathological features were grouped by worst pattern of invasion, depth of invasion, PNI and ENE. Nodal involvement was significantly associated with ENE, with 42 node‐positive cases presenting with positive ENE (Pearson’s *χ* ^2^ = 49.229, *p* < 0.05)_._ Such a breach in the lymph node capsule is more likely to expose the metastatic tumour cells for distant spread.

### 3.5. Association Between the Various Histological Parameters

Depth of invasion was significantly associated with pathological T stage, with deeper invasions noted in advanced stages (*p* < 0.05, Table S1). Cases presenting with pT4 had a deeper front of tumour invasion of more than 10 mm. Clinically, tumour size seems to have a direct bearing on the extent of infiltration, thus having a synergistic bearing on tumour aggressiveness.

A significant correlation was found between the worst pattern of invasion and depth of invasion (*p* < 0.05, Table S2). Higher invasive patterns correlated with greater PNI and deeper invasion, indicating increased tumour aggressiveness.

In both cases of WPOI and DOI, a higher grade for both was found in relation to the presence of PNI around large nerves, indicated by the presence of both DOI > 10 mm as well as Type IV WPOI in a solitary case of PNI around a large nerve. Depth of invasion significantly correlated with ENE, with deeper invasions predictive of extranodal spread (*p* < 0.05, Table 3).

**Table 3:** Association between depth of invasion and extranodal extension in oral squamous cell carcinoma.

Parameter	Status	Extranodal extension		
		Not assessed	Present	Absent
Depth of invasion	Not assessed	5	5	6
	<5 mm	0	13	5
	>5 mm	0	16	5
	>10 mm	1	17	12
	*χ* ^2^	21.465		
	*p*‐Value	0.001513 ^∗^		

*Note*: *p*‐Value was calculated using Pearson’s chi‐square test. A *p* < 0.05 was considered statistically significant.

^∗^Indicates significance.

### 3.6. Kaplan–Meier Survival Analyses

**Figure 1:**
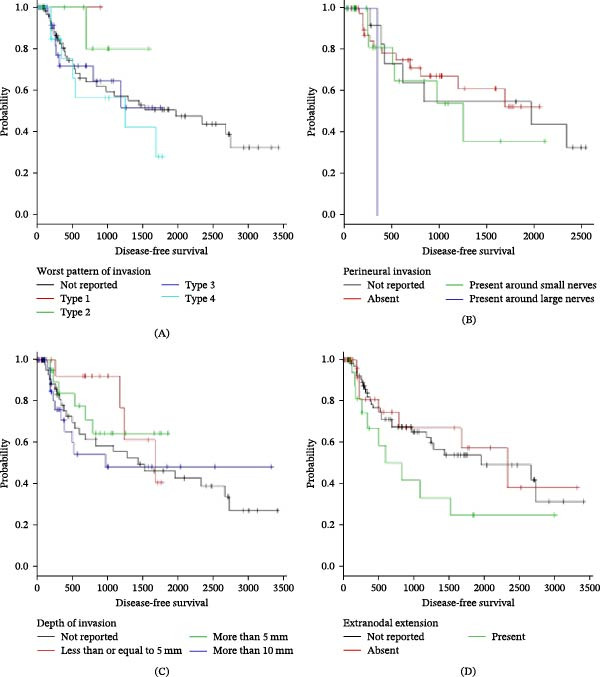
Kaplan–Meier analysis for disease‐free survival stratified by histopathological features. (A) Disease‐free survival according to the worst pattern of invasion (log‐rank *p* = 0.575). (B) Disease‐free survival according to perineural invasion status (log‐rank *p* = 0.308). (C) Disease‐free survival according to depth of invasion (log‐rank *p* = 0.632). (D) Disease‐free survival according to extranodal extension status (log‐rank *p* = 0.663). No statistically significant differences were observed across the groups.

**Figure 2:**
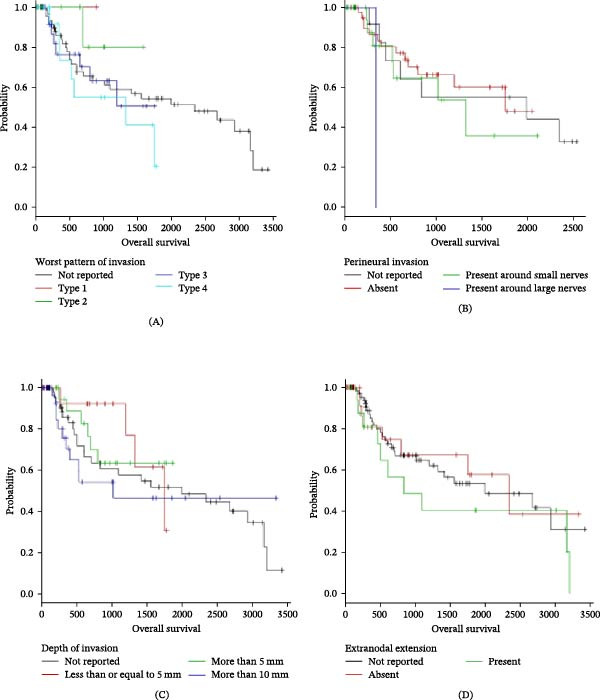
Kaplan–Meier analysis of overall survival stratified by histopathological features. (A) According to the worst pattern of invasion (log‐rank *p* = 0.575). (B) According to perineural invasion status (log‐rank *p* = 0.196). (C) According to depth of invasion (log‐rank *p* = 0.594). (D) According to extranodal extension status (log‐rank *p* = 0.639). No statistically significant differences were observed across the groups.

Kaplan–Meier analyses clearly demonstrated that histopathological features showed trends toward poorer survival outcomes with increased depth of invasion and more aggressive invasion patterns, although statistically non‐significant. The survival curve trend shows a sharp drop in survival probability with every increasing grade of each adverse histological feature. The presence of one or more of these adverse histopathological features, by virtue of its bearing on diminished survival prospects, could alter treatment strategies from a clinical standpoint. (Figures 1 and 2) (Table 4).

**Table 4:** Kaplan–Meier survival analyses of histopathological features in oral squamous cell carcinoma.

Parameter	Status	Disease‐free survival		Overall survival	
		*n*	Median survival	*n*	Median survival
Depth of invasion	Not assessed	16	1965	16	1998
	<5 mm	18	1685	18	1757
	>5 mm	21	NA	21	NA
	>10 mm	30	975	30	1022
	Log‐rank (Mantel Cox)	1.7		1.9	
	*p*‐Value	0.632		0.594	
Worst pattern of invasion	Not assessed	29	1965	29	1998
	Type I	2	NA	2	NA
	Type II	10	NA	10	NA
	Type III	27	NA	27	NA
	Type IV	17	1248	17	1328
	Log‐rank (Mantel Cox)	2.9		2.9	
	*p*‐Value	0.575		0.575	
Perineural invasion	NA	13	1965	13	1998
	A	45	NA	45	1757
	P	26	1248	26	1328
	PL	1	346	1	346
	Log‐rank (Mantel Cox)	3.6		4.7	
	*p*‐Value	0.308		0.196	
Extranodal extension	Not assessed	6	837	6	842
	Absent	51	1965	51	1998
	Present	28	2339	28	2344
	Log‐rank (Mantel Cox)	0.8		0.9	
	*p*‐Value	0.663		0.631	

*Note*: *p*‐Values were calculated by log‐rank method. *p* < 0.05 was considered statistically significant. PL, present around large nerve.

Abbreviations: A, absent; NA, not assessed; P, present.

^∗^Indicates significance.

## 4. Discussion

Risk assessment and patient stratification currently centre around the TNM staging under the 8th Edition of the American Joint Cancer Committee’s Cancer Staging Manual [12]. A sharp drop in disease‐free survival when there is regional lymph node metastasis merits the requirement of more accurate risk prediction models [13]. Currently, primary surgery with curative intent and clear margins serves as the first line of treatment, with adjuvant radiotherapy alone or in combination with chemotherapy planned based on National Comprehensive Cancer Network guidelines [14].

The Brandwein‐Gensler et al.’s [15] histological risk assessment model was able to predict locoregional recurrence and survival by grading certain adverse histological features, namely the pattern of invasion, PNI and lymphocyte host response. Further evidence on more adverse histological features and risk assessment and risk prediction models, including, depth of invasion, lymphovascular invasion and ENE have all been able to predict recurrence and survival with varying degrees of success [16, 17].

In our study, first, a significant association was found among the various histological features. A higher grade of pattern of invasion, especially in Type 4, with tumour islands comprising less than 15 cells per island, was significantly associated with PNI. This finding was in agreement with the work of Brandwein‐Gensler et al. [7, 15]. From a biological viewpoint, it could indicate a role for cellular dyscohesion in the neurotropic behaviour of tumour cells in OSCC.

Yoshizawa et al. [18] in their study, found a significant association between depth of invasion and pattern of invasion. The study assessed the pattern of invasion based on the Yamamoto‐Kohama (YK) classification system [19]. The types 4C and 4D, which correspond to the most dyscohesive pattern in the YK system, closely resemble the WPOI‐4 and WPOI‐5 of the Brandwein‐Gensler classification system, which is used in our study. There was a strong association between a YK4C pattern of invasion with depth of invasion. Similarly, our study also found a strong association between Type 4 WPOI and depth of invasion. Evidently, this may indicate the weakening of cell–cell adhesions as the depth of invasion increases [20].

ENE or extracapsular spread is now a component of the pathological N‐stage criterion of TNM staging [17]. In our study a significant association of ENE with depth of invasion was found. This finding is in line with the study by Bera et al. [21]. The presence of ENE was significantly associated with a depth of invasion of more than 10 mm. This gains significance in cases of occult neck metastasis [22]. A study by Alvi and Johnson [23] found that incidences of ENE can be as high as 49% even in clinically N0 nodes. Thus, a DOI of more than 10 mm could indeed indicate an occult neck metastasis and require a sentinel lymph node biopsy [24].

Our study also showed a significant association between ENE and PNI, but its utility as an independent predictor is questionable since 32 cases of negative PNI cases reported positive ENE, while only 13 cases of positive PNI presented with positive ENE. Existing literature on the association between ENE and PNI is also mixed, with studies by Alzahrani et al. [25] favouring a positive association while a study by Mair et al. [26] points to the contrary. Thus, it is difficult to conclude whether the presence of PNI predisposes regional lymph nodes to exhibit ENE.

Last, a significant association between the depth of invasion and PNI was present as well. The incidence of encountering PNI steadily increased with increasing depth of invasion. Similar findings were also evidenced in the studies by Navarro Cuéllar et al. [27], Rahima et al. [28] and Arun et al. [29]. The neurotropic aspect of OSCC tumour cells and the use of nerve sheaths as a pathway for distant dissemination of tumour cells have also been attributed [30].

In terms of its impact on survival, WPOI, DOI and PNI exhibited a definite trend in predicting overall and disease‐free survival in our study. Higher grades of all three parameters showed a downward trend in survival which was in line with the existing literature. The findings on DOI were in agreement with those of a large multi‐centric cohort study by Ebrahimi et al. [8] which eventually led to the incorporation of DOI in the pathological T‐stage of the 8th AJCC (AJCC 8) Cancer staging. Higher grades of WPOI also pointed towards a downward trend in survival, which was in line with the Brandwein‐Gensler et al.’s [15] histological risk assessment model. PNI as an independent predictor for poor 5‐year OS and DSS, as evidenced by Caponio et al. [31] and Arun et al. [29] was closely replicated in our study as well.

Despite the pathobiological and prognostic implications, the employability of histopathological features as either standalone risk assessment models or their incorporation into an existing risk stratification framework like the AJCC criteria suffers from a few roadblocks, including inter‐observer agreement and variability, setting of cut‐off values, dichotomisation and lack of validation studies on developed models. Of note, PNI, one of the histological features evaluated in this study as well, is seen to display variations in inter‐observer agreement [17]. The variations have been reported either as the definition of PNI itself or the description of the perineurum or perineurium involved. Studies by Batsakis [32] and Liebig et al. [33] define PNI as the presence of tumour cells in close proximity to a nerve involving at least a third of its circumference. However, there is wide variability in the assessment of the proximity of the tumour cells to a nerve to qualify as PNI, with ambiguity regarding the degree of encirclement of the nerve (incomplete or complete) and layers of nerve involved [34]. Subcategorization of PNI on the basis of extent, layer of nerve sheath where tumour cells are present and size and number of nerves also yielded conflicting results, with size of involved nerves showing an inverse correlation with survival [29].

In the case of depth of invasion, studies have sought to differentiate it from tumour thickness and have attempted to assign cut‐off values for its measurement. A study by Dirven et al. [35] found similar prognostic ability for both DOI as well as tumour thickness when it came to modifying the pathological T stage in the 8th TNM staging. Regarding assigning of cut‐off values, Almangush et al. [36] found a cut‐off of 2 mm for pathological T1 and 4 mm for T2 to have a better prognostic ability. While our study employed cut‐off values independent of their role as modifiers in prognostication based on TNM staging, the results as independent prognosticators were close to those by Shinn et al. [37] where they found a cumulative incidence of regional metastasis with increasing depth of invasion.

In the case of ENE, there is a need for a standardised definition as well so as to minimise interobserver variability. Abdel‐Halim et al. [38] in their systematic review propose ENE to be defined as ‘squamous cell carcinoma within the confinement of a metastatic lymph node that grows through the lymph node capsule or beyond the lymph node contour into the adjacent tissue regardless of the size of the extension’. While the AJCC 8th edition prescribes categorising ENE as either being present (positive) or absent (negative) it also gives a subcategorization of positive cases of ENE into ENE_mi_ or microscopic ENE, where extension of tumour outside the lymph node capsule is less than 2 mm and ENE_ma_ or major ENE, where extension is more than 2 mm. But a study by Tirelli et al. [39] found no significant association of the degree of extension of ENE with the survival outcomes.

The trends evidenced in our study indicate a possible prognostic value, although conclusive statistical significance for WPOI, DOI and PNI as independent survival predictors was limited by sample size and follow‐up duration, highlighting the need for further studies.

## 5. Conclusion

To conclude, histological risk assessment and stratification could aid in improving patient outcomes and in planning adequate treatment strategies. Our study indicates that histological risk assessment could complement existing TNM staging criteria or potentially serve as an independent stratification tool. Recently, histological risk assessment models like the Aditi‐Nuzhat Lymph Node Prediction system have shown utility in predicting nodal metastasis [40]. The addition of DOI to pT stage and ENE to pN stage in the AJCC 8th edition of the cancer Staging Manual and its subsequent validation in retrospective cohorts saw an upstaging of several cases, subsequently reflecting on their treatment planning [41]. In light of this, our study, which saw similar trends in the predictive ability of adverse histological features in terms of survival, makes a case for including WPOI in patient risk stratification, possibly as a complement to the pathological T stage criterion. The mutual association between the various adverse histological features found in our study could point to a common underlying pathophysiology.

## Author Contributions

**Shyam Sthanukrishnan**: conceptualisation, data curation, formal analysis, investigation, methodology, writing – original draft, writing – review and editing. **Smitha Sammith Shetty**: data curation, formal analysis, methodology, validation, writing – review and editing. **Chetana Chandrashekar**: conceptualisation, formal analysis, methodology, validation, writing – review and editing. **Deepak Nayak**: data curation, investigation, resources, methodology. **Raghu Radhakrishnan**: conceptualisation, project administration, resources, supervision, funding acquisition, validation, writing – review and editing.

## Funding

This work was supported by the DBT/Wellcome Trust India Alliance Fellowship (Grant IA/CPHI/18/1/503927) awarded to Raghu Radhakrishnan.

## Ethics Statement

The study protocol was approved by the Institutional Ethics Committee of the Kasturba Medical College and Kasturba Hospital, Manipal in October 2023 (Kasturba Medical College and Kasturba Hospital Institutional Ethics Committee Approval Number 506/2023).

## Conflicts of Interest

The authors declare no conflicts of interest.

## Supporting Information

Additional supporting information can be found online in the Supporting Information section.

## Supporting information


**Supporting Information** Table S1: Association between histopathological features and AJCC 8th Edition Pathological TNM Staging. Table S2: Association between histopathological features.

## Data Availability

Data are available from the corresponding author upon reasonable request.
