# Supplementary material for: Survival Trends Associated With Histopathological Risk Factors in Oral Squamous Cell Carcinoma
Source: Int J Dent. 2026 May 8;2026:8821799. doi: 10.1155/ijod/8821799 (PMC13155408; doi:10.1155/ijod/8821799)
Supplement: Supplementary file 1 — Supporting Information Table S1: Association between histopathological features and AJCC 8th Edition Pathological TNM Staging. Table S2: Association between histopathological features. [file IJOD-2026-8821799-s001.docx]

**Supplementary Table 1:** Association between histopathological features and AJCC 8^th^ Edition Pathological TNM Staging in oral squamous cell carcinoma

| Parameter | Status | pT stage | | | | pN stage | | | |
| --- | --- | --- | --- | --- | --- | --- | --- | --- | --- |
|  |  | **1** | **2** | **3** | **4** | **0** | **1** | **2** | **3** |
| DOI | **NA** | 3 | 6 | 1 | 6 | 7 | 4 | 1 | 4 |
|  | **<5mm** | 8 | 6 | 1 | 3 | 11 | 1 | 2 | 4 |
|  | **>5mm** | 0 | 17 | 1 | 3 | 11 | 3 | 3 | 4 |
|  | **>10mm** | 2 | 1 | 1 | 6 | 15 | 1 | 3 | 11 |
|  | ***χ^2^*** | 54.974 | | | | 8.1257 | | | |
|  | *p*-value | 0.00000001232* | | | | 0.5215 | | | |
| WPOI | **NA** | 5 | 10 | 5 | 9 | 13 | 5 | 6 | 5 |
|  | **Type I** | 2 | 0 | 0 | 0 | 2 | 0 | 0 | 0 |
|  | **Type II** | 2 | 2 | 3 | 3 | 8 | 0 | 1 | 1 |
|  | **Type III** | 3 | 13 | 2 | 9 | 13 | 2 | 1 | 11 |
|  | **Type IV** | 1 | 5 | 4 | 7 | 8 | 2 | 1 | 6 |
|  | *χ^2^* | 18.078 | | | | 15.13 | | | |
|  | *p*-value | 0.1133 | | | | 0.2344 | | | |
| PNI | **NA** | 4 | 4 | 1 | 4 | 7 | 4 | 1 | 1 |
|  | **A** | 8 | 18 | 8 | 11 | 26 | 3 | 5 | 11 |
|  | **P** | 1 | 8 | 5 | 12 | 10 | 2 | 3 | 11 |
|  | **PL** | 0 | 0 | 0 | 1 | 1 | 0 | 0 | 0 |
|  | *χ^2^* | 10.147 | | | | 12.292 | | | |
|  | *p*-value | 0.3387 | | | | 0.1973 | | | |
| ENE | **NA** | 1 | 1 | 0 | 4 | 3 | 3 | 0 | 0 |
|  | **Absent** | 11 | 17 | 8 | 15 | 41 | 5 | 4 | 1 |
|  | **Present** | 1 | 12 | 6 | 9 | 0 | 1 | 5 | 22 |
|  | ***χ^2^*** | 8.6304 | | | | 76.056 | | | |
|  | *p*-value | 0.1955 | | | | 2.327e-14* | | | |

*p*-values were calculated using Pearson’s Chi-Square test. *p* < 0.05 was considered significant. * indicates significance.

NA - Not Assessed. pT - Pathological T-stage; pN - Pathological N-stage; DOI - Depth of invasion; WPOI - Worst pattern of invasion; PNI - Perineural invasion; ENE- Extranodal extension.

**Supplementary Table 2:** Association between depth of invasion, extranodal extension and perineural invasion in oral squamous cell carcinoma

| Parameter | Status | Worst pattern of invasion | | | | | Perineural Invasion | | | |
| --- | --- | --- | --- | --- | --- | --- | --- | --- | --- | --- |
|  |  | **NA** | **Type I** | **Type II** | **Type III** | **Type IV** | **NA** | **P** | **PL** | **A** |
| Depth of invasion | **NA** | 15 | 0 | 0 | 0 | 1 | 12 | 3 | 0 | 1 |
|  | **<5mm** | 1 | 2 | 6 | 6 | 3 | 0 | 2 | 0 | 16 |
|  | **>5mm** | 3 | 0 | 1 | 11 | 6 | 0 | 8 | 0 | 13 |
|  | **>10mm** | 10 | 0 | 3 | 10 | 7 | 1 | 13 | 1 | 15 |
|  | *χ^2^* | 51.286 | | | | | 64.193 | | |  |
|  | *p*-value | 0.0000008292* | | | | | 2.07e-10* | | |  |
| Extranodal Extension | **NA** | 6 | 0 | 0 | 0 | 0 | 5 | 0 | 0 | 1 |
|  | **P** | 13 | 2 | 8 | 17 | 11 | 5 | 13 | 1 | 32 |
|  | **A** | 10 | 0 | 2 | 10 | 6 | 3 | 13 | 0 | 12 |
|  | *χ^2^* | 15.355 | | | | | 27.738 | | | |
|  | *p*-value | 0.05259 | | | | | 0.0001053* | | | |
| Perineural Invasion | **NA** | 13 | 0 | 0 | 0 | 0 | - | - | - | - |
|  | **A** | 11 | 2 | 7 | 15 | 10 | - | - | - | - |
|  | **P** | 5 | 0 | 3 | 12 | 6 | - | - | - | - |
|  | **PL** | 0 | 0 | 0 | 0 | 1 | - | - | - | - |
|  | *χ^2^* | 35.989 | | | | | | | | |
|  | *p*-value | 0.0003253* | | | | | | | | |

*p*-values were calculated using Pearson’s Chi-Square test. *p* < 0.05 was sconsidered statistically significant. * indicates significance.

NA - Not Assessed; P - Present; A - Absent; PL - Present around large nerves
